# Quantification of Electromechanical Coupling to Prevent Inappropriate Implantable Cardioverter-Defibrillator Shocks

**DOI:** 10.1016/j.jacep.2019.01.025

**Published:** 2019-06

**Authors:** Daniel Keene, Matthew J. Shun-Shin, Ahran D. Arnold, James P. Howard, David Lefroy, D. Wyn Davies, Phang Boon Lim, Fu Siong Ng, Michael Koa-Wing, Norman A. Qureshi, Nick W.F. Linton, Jaymin S. Shah, Nicholas S. Peters, Prapa Kanagaratnam, Darrel P. Francis, Zachary I. Whinnett

**Affiliations:** a Department of Cardiology, Imperial College Hospitals National Health Service Trust, London, United Kingdom; b National Heart and Lung Institute, Imperial College London, London, United Kingdom

**Keywords:** arrhythmia discrimination, hemodynamic monitoring, implantable cardioverter-defibrillator, inappropriate therapy, laser Doppler perfusion monitoring, AR, arrhythmogenic right, ECG, electrocardiogram, EGM, electrogram, HCM, hypertrophic cardiomyopathy, ICD, implantable cardioverter-defibrillator, IHD, ischemic heart disease, LDPM, laser Doppler perfusion monitoring, LVC, left ventricular cardiomyopathy, LVSD, left ventricular systolic dysfunction, RV, right ventricular, SVT, supraventricular tachycardia, VF, ventricular fibrillation, VT, ventricular tachycardia

## Abstract

**Objectives:**

This study sought to test specialized processing of laser Doppler signals for discriminating ventricular fibrillation (VF) from common causes of inappropriate therapies.

**Background:**

Inappropriate implantable cardioverter-defibrillator (ICD) therapies remain a clinically important problem associated with morbidity and mortality. Tissue perfusion biomarkers, implemented to assist automated diagnosis of VF, sometimes mistake artifacts and random noise for perfusion, which could lead to shocks being inappropriately withheld.

**Methods:**

The study tested a novel processing algorithm that combines electrogram data and laser Doppler perfusion monitoring as a method for assessing circulatory status. Fifty patients undergoing VF induction during ICD implantation were recruited. Noninvasive laser Doppler and continuous electrograms were recorded during both sinus rhythm and VF. Two additional scenarios that might have led to inappropriate shocks were simulated for each patient: ventricular lead fracture and T-wave oversensing. The laser Doppler was analyzed using 3 methods for reducing noise: 1) running mean; 2) oscillatory height; and 3) a novel quantification of electromechanical coupling which gates laser Doppler relative to electrograms. In addition, the algorithm was tested during exercise-induced sinus tachycardia.

**Results:**

Only the electromechanical coupling algorithm found a clear perfusion cut off between sinus rhythm and VF (sensitivity and specificity of 100%). Sensitivity and specificity remained at 100% during simulated lead fracture and electrogram oversensing. (Area under the curve running mean: 0.91; oscillatory height: 0.86; electromechanical coupling: 1.00). Sinus tachycardia did not cause false positive results.

**Conclusions:**

Quantifying the coupling between electrical and perfusion signals increases reliability of discrimination between VF and artifacts that ICDs may interpret as VF. Incorporating such methods into future ICDs may safely permit reductions of inappropriate shocks.

Forty percent of implantable cardioverter-defibrillator (ICD) shocks in clinical practice are inappropriate 1, 2, that is, they are delivered in the absence of a ventricular arrhythmia episode. These cause unpleasant symptoms, increase health care costs 2, 3, 4, and are associated with increased mortality 4, 5, 6. Despite increasingly sophisticated electrogram (EGM) analysis algorithms, it has not been possible to reduce the rate of inappropriate shocks below ∼3% per year, even in carefully recruited, attentively managed clinical trial patients 7, 8. Rates appear to be even higher in clinical practice, affecting up to 41% of ICD recipients (9).

Several situations can be misinterpreted as extreme high rates requiring a shock. T-wave oversensing can cause a 2-fold increase in the apparent heart rate. Right ventricular lead fracture can produce electrical noise artifacts, detected as high rates. Electromagnetic interference can similarly be misdetected as a high heart rate. Occasionally, sinus tachycardia, occurring during exercise, can also lead to inappropriate therapies, particularly in patients with a subcutaneous ICD 10, 11.

If more than a small fraction of shocks are inappropriate, there is room for improvement. The development of new EGM-based algorithms has allowed inappropriate therapy rates for subcutaneous ICDs to be lowered to the same ∼40% at which transvenous ICDs have plateaued 1, 2. Therefore, for all types of implantable defibrillators, there remains a major opportunity to reduce inappropriate therapies in order to make ICDs safer and more acceptable to people at risk of sudden cardiac death.

A potential method for improving on purely EGM-based detection is the addition of information for hemodynamic status. Ventricular fibrillation (VF) inevitably causes hemodynamic collapse; therefore, the presence of good cardiac output eliminates the possibility of VF, even if the EGM-based electrical measurements suggest this. However, if a hemodynamic measurement is to be incorporated into the ICD diagnostic algorithm, it is essential that it provide both a rapid and an extremely reliable assessment of hemodynamic status. This is of key importance to ensure therapies are, first, not inappropriately withheld and, second, to avoid delays in the delivery of lifesaving therapies for true VF.

### Neither steady state (running mean) level nor oscillatory height has proved reliable

Regardless of the hemodynamic marker chosen, reliability of decision making is limited by the common theme of how to process the numerical signal to arrive at a diagnostic classification. There have been 2 approaches investigated for processing hemodynamic markers for this purpose: the running mean approach and the oscillatory height method.

In the running mean approach, an average value for the marker over several seconds is compared to an average value taken from a recent reference time, commonly immediately previously (12). This requires continuous monitoring, which impairs battery longevity. A subtler limitation is that changes in posture, spontaneous biological variations, and movement artifacts can lead to changes in the signal that have no clinical meaning but can be difficult to distinguish from changes caused by arrhythmia.

The second approach is the oscillatory height approach, which quantifies the amplitude of peak-to-trough variation in the hemodynamic signal, as this reduces or eliminates the problem of gradual baseline drift that plagues the running mean approach 13, 14, 15. However, in patients, there are always small movements and disturbances whose frequency and amplitude can overlap those elicited by cardiac function and therefore cannot be reliably removed using standard filtering of the hemodynamic signal alone. As a result, although carefully designed animal experiments showed good discrimination 13, 14, 15, imperfect rapid reliability in humans has prevented clinical application.

### Electromechanical coordination as the signal

This study investigated a new way to process perfusion signals in order to deliver a reliable assessment of hemodynamic status when the apparent EGM heart rate is in the VF zone, to determine whether a shock is required or can be safely withheld. This method combines simultaneous EGM and hemodynamic measurement to confirm whether observed hemodynamic pulsatility is consistent with the apparent electrical rhythm at that instant.

Specifically, the study tested whether the novel algorithm in combination with laser Doppler perfusion signals could reliably assess hemodynamic status during VF and during sinus rhythm, even in the presence of double-counting, simulated lead fracture, and sinus tachycardia. We compared the reliability of this novel algorithm as compared to those of the previously described running mean and oscillatory height approaches.

## Methods

### Study population

Patients who were undergoing clinically planned VF induction during clinically indicated ICD implantation were recruited. VF was induced with the patients under general anesthesia for those with subcutaneous ICDs and under intravenous sedation for those with transvenous ICDs, using a 50-Hz burst or a drive train and T-wave synchronized shock.

### Measurements

Patients were continuously monitored using beat-by-beat blood pressure. Where invasive monitoring was not clinically indicated, this was done noninvasively (Finapres Nova, Finapres Medical Systems, Enschede, the Netherlands). Patients had 2 laser Doppler sensors (Periflux 5000, Perimed, Järfälla, Sweden) positioned noninvasively on the skin, 1 on the index finger of the hand contralateral to the implant and the other on the chest wall (Online Figure 1). Patients also had continuous electrocardiographic (ECG) monitoring (Fukuda Denshi 7100, Fukuda Denshi, Tokyo, Japan).

Where possible, the EGM from the ICD lead and the device marker channel through the Pace-Sense Analyser were simultaneously acquired. In the remaining participants, the surface ECG was used as a substitute for the ICD lead signal.

Recording began at least 1 min before VF induction and continued until at least 1 min after returning to sinus rhythm. Data were acquired at 1,000 Hz using an analog-to-digital card (National Instruments, Austin, Texas) and Labview (National Instruments). Signals were then analyzed off-line, using automated customized software (Python Foundation, Wilmington, Delaware).

### Sampling windows

**Figure 1:**
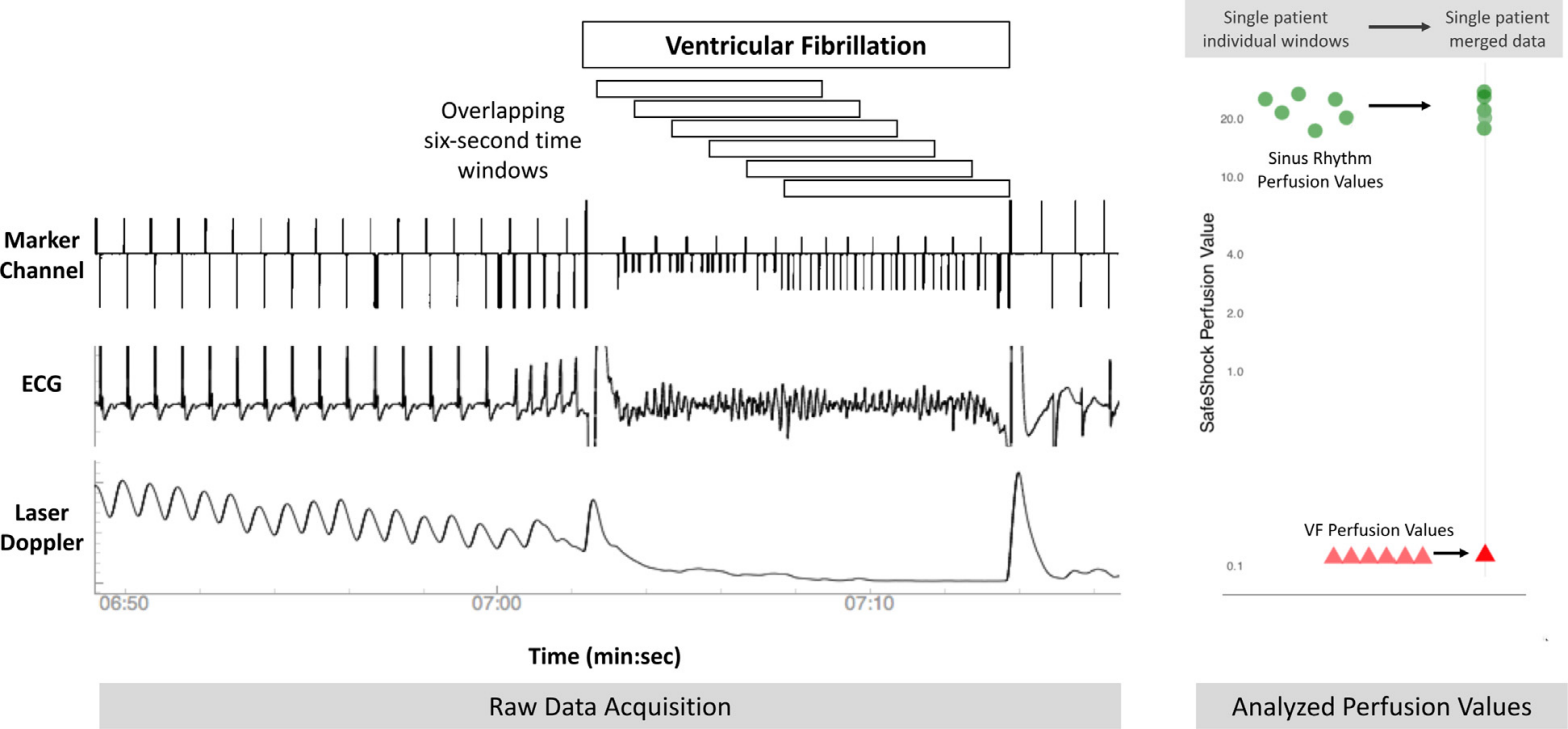
Data Sampling Windows **(Left)** For each VF episode, overlapping 6-s time windows were analyzed. (The same number of sinus rhythm windows were analyzed from immediately before VF induction.) **(Right)** For each window, a perfusion value was calculated (**green circles** for sinus rhythm, **red triangles** for VF). VF = ventricular fibrillation.

It was important to ensure that test results could not be unintentionally manipulated through the authors’ selection of the time window during which the algorithm ran. Therefore, each VF episode was analyzed as a series of overlapping 6-s time window, starting from the onset of VF and stepping forward by 1 s at a time until the end of the period of VF (Figure 1). Similarly, the sinus rhythm analysis was predefined so that windows would be the same number of overlapping 6-s window but from immediately before VF induction and working backward. This meant that for each episode there were multiple (at least 4) individual measurements during VF and that there were the same number during sinus rhythm. All measurements were included in the analysis.

### Choice of hemodynamic sensor

Beat-by-beat cardiac output or blood pressure may be seen as ideal methods for assessing clinical status. However, long-term monitoring of systemic arterial blood pressure or cardiac output is not straight forward because intra-arterial devices can be difficult to implant, risk thrombosis, require separate power, and need to communicate with the ICD.

An alternative is to assess perfusion. Methods for this include photoplethysmography, near infrared spectroscopy, and laser Doppler perfusion monitoring. These methods rely on various properties of reflected light rather than on a pressure sensor inside the blood stream. Some studies have previously been conducted to investigate an approach for augmenting ICD discrimination 12, 13, 14, 15.

### Laser doppler perfusion monitoring

Laser Doppler quantifies flow from the change in wavelength of a laser signal reflected back from moving red blood cells (16). Pulsatility of blood flow is then detected (Online Figure 1) 17, 18, 19.

### Datasets for 4 conditions per patient

Recordings were made for each patient during sinus rhythm and VF. The EGM recorded during sinus rhythm was also transformed to simulate lead fracture and double-counting.

To simulate lead fracture, the ventricular signal in each patient was replaced with an EGM from a clinical lead fracture. The algorithm was then supplied with the atrial lead’s sensed electrical data or, if this was unavailable, the original ventricular lead data was used but shifted backward by an appropriate atrioventricular delay (120 ms).

To simulate double-counting, an extra sensed R-wave was synthesized between the truly detected R-waves at 33% of the R-R interval. During processing with the novel algorithm, the new approach formed multiple hypotheses about which R-waves were true or false and tested them against the perfusion signal.

### Sinus tachycardia data set

To test misdetection of sinus tachycardia, heart rates were required in the range of 180 to 200 beats/min. This required a different group of subjects. We recruited 12 healthy volunteers, who exercised on a treadmill to a peak heart rate that averaged 184 beats/min. ECG, laser Doppler, and noninvasive beat-by-beat blood pressure values were recorded.

### Analytical algorithm

The novel electromechanical coupling algorithm has previously been described in detail (20). Briefly, it begins with 3 simultaneous analyses of the laser Doppler data, each with a different gating. The primary gating is with the ventricular lead, the secondary is with an alternate lead or vector, and the third is with a synthetic signal designed to deal with overcounting.

It is currently designed to be used as a confirmatory step after conventional EGM discriminators. It is envisaged that the laser Doppler assessment would only need to be activated once the conventional EGM algorithms have started to detect suspected VF. If there is sufficient pulsatility (exceeding a global threshold which does not need personalizing to the patient) with the primary gating, the algorithm reports this and halts. If there is not sufficient pulsatility, the algorithm tests for the possibility of disruption by noise (by trying the secondary gating) or disruption by oversensing (by trying the tertiary gating). If the secondary or tertiary gating reveals satisfactory pulsatility, the algorithm reports this, otherwise it reports insufficient pulsatility. Although these tests are described in sequence, they are actually conducted simultaneously (Online Figure 2). If conventional EGM algorithms continue to suspect VF, the laser Doppler quantification can be repeated as often as required. Additionally, the laser Doppler assessment can detect lead fracture from the difference between the results of primary and secondary gating. This could be used to alert the patient’s medical team.

This analysis focused on the algorithm in its 3 component parts: 1) the primary gating; 2) the secondary gating with an alternate electrode (to deal with lead fracture); and 3) the tertiary gating with an alternate EGM interpretation (for oversensing).

The perfusion data were also analyzed using 2 previously described algorithms. The first algorithm calculates the running mean of the perfusion data over the analytical window (12). The second calculates the mean peak-to-trough height after passing a band-pass filter over the analytical window (the oscillatory height method) 13, 14, 15. No absolute cutoff values have been described for these previously published methods.

Quantification of electromechanical coupling, the running mean algorithm, and the oscillatory height algorithm were tested for their ability to distinguish between VF and the situations that could mimic VF.

### Statistical analysis

Pilot testing had indicated the electromechanical coupling perfusion value, which runs on a scale from 0 to 100, always has values <2 during VF and always has values above 2 during sinus rhythm. Therefore, this study used a threshold of 2. Sensitivities and specificities were calculated using appropriate methods. The perfusion data were processed using Python software (Python Foundation, Wilmington, Delaware), and statistical analyses were performed using R version 3.0.2 (R Project, Vienna, Austria).

### Study conduct

Patients gave written informed consent for this study, which was approved by the local Research Ethics Committee (14/LO/2158).

## Results

### Patient population

Fifty patients were recruited (Table 1). Nineteen patients were having implants for secondary prevention of ventricular arrhythmias and 31 for primary prevention. Thirty-five patients received transvenous devices, and 15 had subcutaneous devices. Twenty-four patients had left ventricular impairment due to ischemic heart disease. The mean age was 61.6 ± 15.5 years, and the mean left ventricular ejection fraction was 39 ± 14.3%). Induction was triggered by 50-Hz bursts in half the patients and by drive train and T-wave shock in the others.

**Table 1:** Patient Demographics and Device Characteristics

Age, yrs	61.6 (±15.5)
Sex	
Male	37 (74)
Female	13 (26)
Ethnicity	
Caucasian	31 (62)
Asian	12 (24)
Afro-Caribbean	7 (14)
Left ventricular ejection fraction, %	39 (±14.3)
Rhythm	
Sinus	48 (96)
Atrial fibrillation	2 (4)
Prevention	
Primary	31 (62)
Secondary	19 (38)
Indication	
LVSD with IHD	24 (48)
LVSD without IHD	10 (20)
HCM	5 (10)
Brugada	2 (4)
Sarcoid	1 (2)
Amyloid	1 (2)
Idiopathic VF	2 (4)
AR+/LVC	5 (10)
ICD Type	
Transvenous	35 (70)
Single-chamber	2 (4)
Dual-chamber	7 (14)
CRT-D	26 (52)
Subcutaneous	15 (30)
Induction method	
R-on-T	25 (50)
50Hz	25 (50)

Values are mean ± SD or n (%).

AR+/LVC = arrhythmogenic right / left ventricular cardiomyopathy; HCM = hypertrophic cardiomyopathy; IHD = ischemic heart disease; LVSD = left ventricular systolic dysfunction.

### Test 1: Confirming onset of VF using continuous doppler measurement

A rudimentary test of discriminative power for the 3 processing algorithms (running mean, oscillatory height, and quantification of electromechanical coupling) is to assess the ability to correctly detect the change from sinus rhythm to VF on VF induction, assuming a continuous stream of Doppler data is being acquired. This test was considered rudimentary because it assumes there is sufficient energy to perform continuous Doppler measurements so that the algorithms always have an immediately preceding segment of normal rhythm against which to compare the suspected VF.

For each patient, the algorithms have the data from 4 or more overlapping windows of VF and the same number of immediately preceding windows of sinus rhythm. In this section of the analysis, for an algorithm to be counted as successful in a patient, it is only required for there to be no overlap between the values in VF and those in sinus on that patient. This method mimics a mode of implementation where there is continuous laser Doppler measurement so that when the algorithm is triggered, there is always an immediately prior reference. This way, the threshold is individualized not only to the patient but also to the particular episode of suspected VF since the immediately preceding normal rhythm is available for reference.

**Figure 2:**
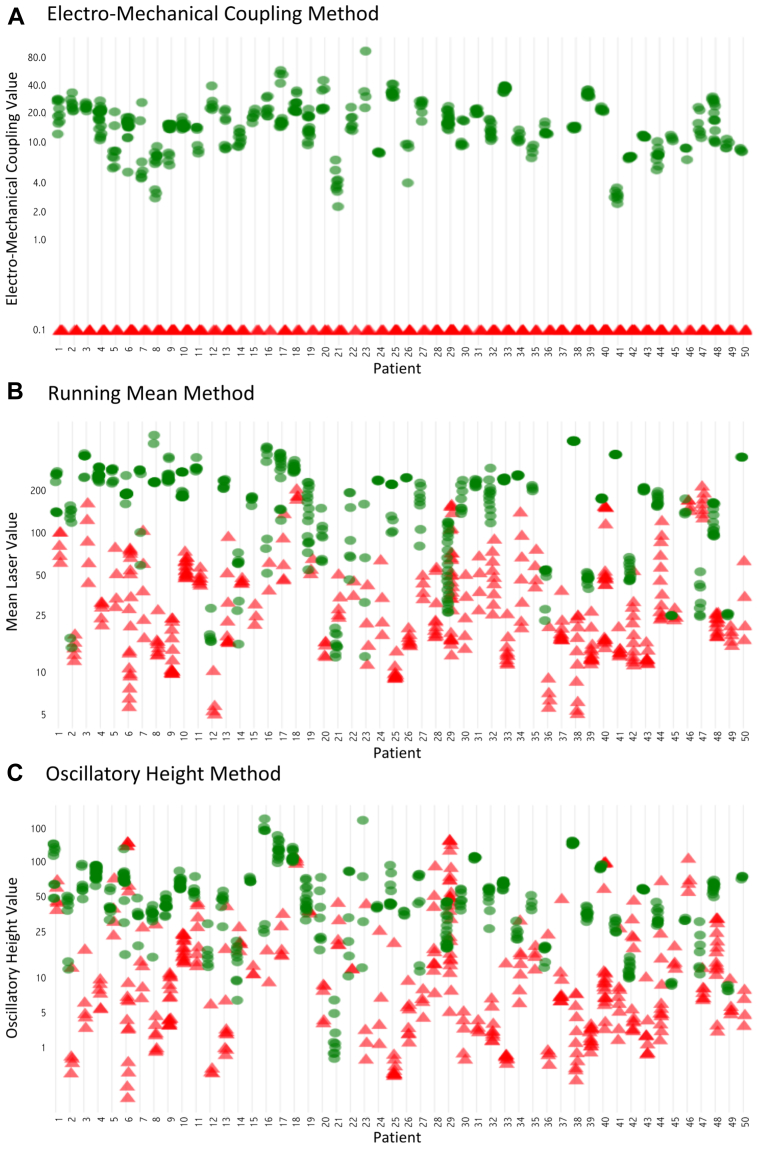
Discriminative Capability of Laser Doppler to Differentiate Sinus Rhythm From Ventricular Fibrillation Perfusion values are shown as **green circles** (sinus rhythm windows) and **red triangles** (VF windows). **(A)** The Electro-Mechanical coupling method shows 100% discrimination for each patient between the 2 states. No sinus rhythm window of any patient scored <2. **(B)** Running Mean method, failing to discriminate in 12 patients. **(C)** Oscillatory Height method, failing in 26 patients.

The running mean was successful in this analysis in 76% of patients (38 of 50). Oscillatory height was successful in 48% (24 of 50) of patients, which was significantly lower (p = 0.004). Electromechanical coupling was successful in 100% (50 of 50) of patients, which was significantly higher than both running mean (p = 0.0002) and oscillatory height (p < 0.0001) (Figure 2).

### Test 2: Confirming onset of VF without continuous doppler measurement

In practice, it would be preferable that the laser would only be switched on when required, to conserve the device’s battery life. This means the algorithm is initiated only to confirm an EGM suspicion of VF; there would be no recording of the immediately preceding normal rhythm. Therefore, the algorithm would work most effectively if there was a complete separation between all the values in sinus rhythm (in all patients) and all the values in VF (in all patients), so that a common discriminatory threshold could be used.

The full spectrum of potentially discriminatory thresholds was tested. Each window of VF was treated individually, yielding 393 VF windows from the 50 patients. Similarly, each of the 393 sinus rhythm windows was treated individually. For every possible discriminatory threshold, a sensitivity and specificity level were calculated across all of these windows and plotted on a receiver-operating characteristic curve.

**Figure 3:**
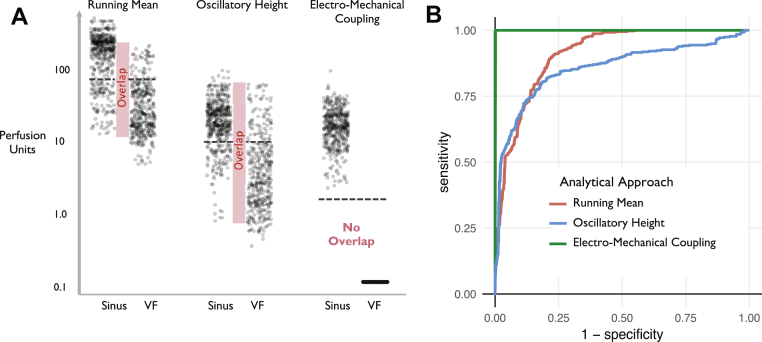
Discriminative Ability of the Running Mean, Oscillatory Height, and Electromechanical Coupling Methods **(A) Horizontal gray dotted lines** indicate thresholds for each analytical mode, chosen to maximize the sum of sensitivity and specificity. The Running Mean and Oscillatory Height had substantial overlap between sinus rhythm and ventricular fibrillation (VF) perfusion data. **(B)** Receiver-operating characteristic (ROC) curves for Running Mean, Oscillatory Height and Electro-Mechanical coupling analytical methods. The area under the curves are 0.91, 0.86, and 1.00, respectively.

Running mean provided an area under the curve (AUC) of 0.91. Oscillatory height provided an AUC of 0.86, which was significantly lower (p < 0.0001). The Electro-Mechanical Coupling algorithm provided an AUC of 1.00, which was significantly higher than both the running mean (p < 0.0001) and the oscillatory height (p < 0.0001) (Figure 3B). Examples of where the running mean and oscillatory height methods fail are shown in Online Figure 3.

### Test 3: Correct handling of ventricular lead fracture

When the ventricular lead develops a fracture, artifacts can occur at a frequency which falls in the VF zone. Even the presence of a normal rate in an alternate lead (i.e., the atrium) does not provide adequate reassurance as VF can coexist with normal atrial activity. The electromechanical coupling approach includes a specific test of hemodynamic pulsatility in synchrony with an alternate lead (or an alternate EGM source, e.g., a sensor located in the generator), so that it can distinguish between a fracture of the ventricular lead (in which situation there will be preserved hemodynamic pulsatility in synchrony with the alternate EGM) and VF (where there will not, even if there is normal atrial activity).

In this test, the EGM signal from a fractured lead was imposed in place of the true ventricular lead signal during sinus rhythm. Each of the 3 algorithms were triggered and tested whether they categorized the situation as VF or not. This was carried out with data from each of the 50 patients.

For each algorithm, it was planned to apply the threshold derived from the sinus rhythm and VF data in test 2 (Figure 3A). The threshold was chosen to maximize the sum of sensitivity and specificity. In practice, electromechanical coupling showed such a large separation between the VF and sinus rhythm data in test 2 that any threshold between 0.1 and 2 perfusion units provided optimal results (both sensitivity and specificity were 100%). Therefore the value of 2 perfusion units derived from previous pilot experiments was used as the electromechanical coupling threshold in test 3.

The running mean (threshold: 80 perfusion units) correctly categorized the lead fracture windows in 52% (26 of 50) patients and oscillatory height (threshold: 10 perfusion units) in 62% (31 of 50) patients. Electromechanical coupling correctly categorized the lead fracture windows in 100% (50 of 50) patients, which was significantly higher than both the running mean (p < 0.0001) and the oscillatory height (p < 0.0001).

### Test 4: Correct handling of electrogram double-counting

Devices are susceptible, despite blanking algorithms, to picking up extra parts of the cardiac cycle beyond the intended activation, for example, the T-wave, and thereby resulting in a 2-fold overestimation of the heart rate. The electromechanical coupling algorithm tests for this possibility, by quantifying the pulsatility not only with a full-set of the suspected R-waves but also with subsets that make allowances for EGM double-counting. If it finds satisfactory pulsatility in association with 1 or more of these subsets, it can advise that this is not VF.

In test 4, each algorithm was given laser Doppler data from sinus rhythm but paired with a modified version of the corresponding electrical data. The modification implemented T-wave double-counting.

The running mean (threshold: 80 perfusion units) correctly categorized the double-counting windows in 52% (26 of 50) patients and oscillatory height (threshold: 10 perfusion units) in 62% (31 of 50) patients. SafeShock correctly categorized the double-counting windows in 100% (50 of 50) patients, which was significantly higher than both the running mean (p < 0.0001) and the oscillatory height (p < 0.0001).

### Test 5: Behavior of algorithms during physical activity

Sometimes, in young, fit individuals, sinus tachycardia can reach the rate of the VF zone. Hemodynamic signals can themselves be vulnerable to increased noise during exercise. We tested the ability of the 3 algorithms to correctly interpret the laser Doppler signals in 12 volunteers exercising to a mean maximum heart rate of 184 beats/min (maximum: 201 beats/min).

For each individual, 6-s windows around the peak heart rate were analyzed, and no electromechanical coupling quantification perfusion value was below the cutoff of 2 perfusion units. The Central Illustration shows how the electromechanical algorithm behaves in various situations.

**Central Illustration:**
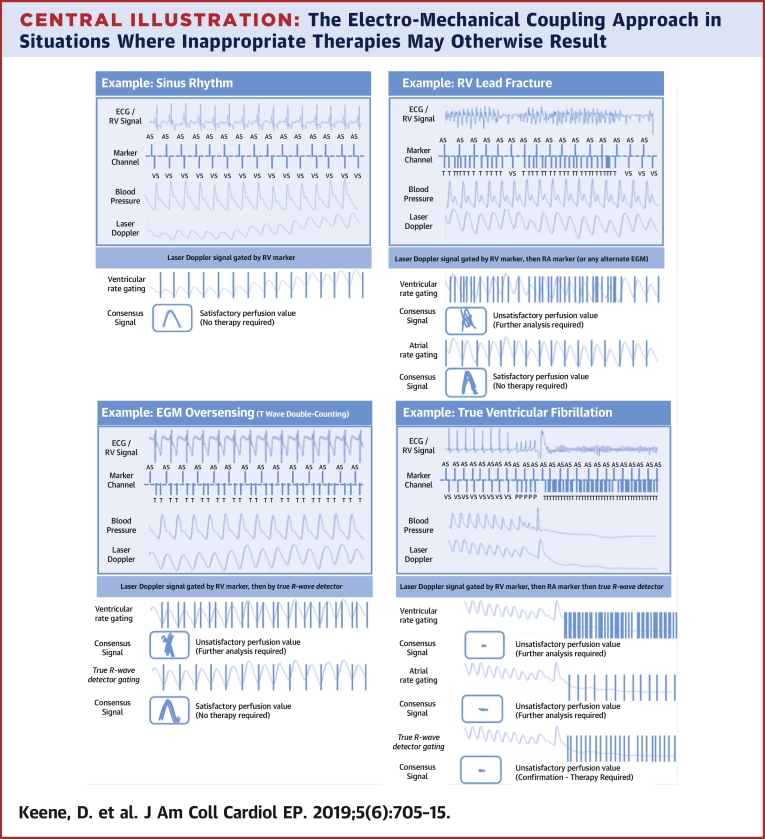
The Electro-Mechanical Coupling Approach in Situations Where Inappropriate Therapies May Otherwise Result Sinus Rhythm: Gating the laser Doppler signal by the R-R interval from the right ventricular (RV) lead shows a satisfactory consensus perfusion value. A similar situation would occur in other well tolerated rhythm disturbances. RV lead fracture: Gating the laser Doppler signal by the R-R interval from the fractured RV lead shows an unsatisfactory perfusion value. When gating is performed by an alternate electrical signal (atrial lead in this example) a satisfactory consensus perfusion value is seen. EGM oversensing: Gating the laser Doppler signal by the R-R interval from the RV lead shows an unsatisfactory perfusion value. Appropriate gating by the algorithm (which simultaneously tests multiple hypotheses as to which are the true R waves) shows a satisfactory consensus perfusion value. True VF: Gating the laser Doppler signal by R-R intervals detected by the ICD lead, alternate electrical signal and by the multiple hypothesis method each time reveals no satisfactory perfusion. ECG = electrocardiogram; EGM = electrogram; ICD = implantable cardioverter-defibrillator; VF = ventricular fibrillation.

## Discussion

In this study, it was found that laser Doppler perfusion monitoring can reliably distinguish between genuine VF and a variety of situations that can be mistaken by a device for VF including ventricular lead fracture, EGM double-counting, and sinus-tachycardia. The key to making the diagnostic separation reliable appears to be the quantification of coupling between electrical and hemodynamic data.

### Opportunity and challenge of hemodynamic monitoring

Although hemodynamic monitoring is potentially an ideal method of augmenting the reliability of EGM-based event discrimination, the early suggested potential has not yet turned into clinical reality. One possible reason suggested by the present data is that small biological fluctuations that continue to occur in the early seconds of VF can be mistaken, in the running mean and oscillatory height methods, for meaningful perfusion. (Once VF has persisted for sufficient time, these methods, however, should eventually recognize lack of perfusion, as found by Compton et al. (12).) Our study, which incorporates electromechanical coupling quantification, means a confident automated diagnosis can be reached quickly. Early diagnosis and avoidance of even modest error rates are valuable for ICD algorithms dealing with VF.

Although laser Doppler is a good marker to measure, the reason for its success in the present study was not the choice of marker but the choice of analytical algorithm. Both the running mean and the oscillatory height methods are attractive in principle but have weaknesses in practice that prevent ICDs relying on them for life or death decisions.

The running mean (12) method aims to reduce the impact of noise by averaging a number of beats and comparing this to a reference. However, variables such as laser Doppler flow vary enormously among individuals and over time, so there is no global threshold that can be applied uniformly. The only chance for a running mean algorithm with laser Doppler flow would be with continuous monitoring so that dramatic declines can be detected from any baseline level. The present study indicates that, even at the power consumption cost of continuous monitoring and even with the patient kept stationary, there is unfortunately overlap between sinus rhythm and VF.

Oscillatory height is perhaps a more advanced analytical approach that has the advantage of looking for fluctuations which are always present in a pulsatile cardiovascular system. However, many other sources can also produce fluctuations in hemodynamic markers. The influence of some of these sources can be eliminated by filtering, because their frequency is outside the plausible range of heart rate. Unfortunately, many influences lie within the frequency range of plausible heart rates and therefore are persistent sources of misclassification of VF as normal rhythm.

The third approach, quantification of electromechanical coupling, maximizes the diagnostic information from hemodynamic signals because it automatically focuses on oscillations that are not merely at a plausible rate but are at the exactly correct rate on a beat-by-beat basis.

### Minimization of battery usage

Quantification of electromechanical coupling not only provides more reliable separation between sinus rhythm and VF within individual patients (test 1) but also retains this separation even when all data are pooled across all patients (test 2). This allows a common threshold to be applied across all these patients without personalization. This has an important consequence for battery energy usage, namely that the laser Doppler only needs to be switched on from the time that an EGM is suggestive of VF. This allows the algorithm to have a very low impact on battery longevity.

### Potential to reduce inappropriate therapies

Because 40% of ICD shocks are inappropriate, better discrimination is clinically very desirable but only if it can be done safely.

Laser Doppler perfusion monitoring together with quantification of electromechanical coupling allows many false positive EGM-based VFs to be detected that would facilitate shocks to be safely withheld. Sensitivity and specificity were 100%, and there was a wide margin of safety, not only within individual patients but also when the data of all patients were merged (i.e., without personalization). The data in this study are encouraging and, the authors believe, provide justification for further prospective testing.

### Hemodynamic sensor within an ICD

Laser Doppler sensors have recently been miniaturized to a size that could be incorporated into a future ICD generator design. Our study made perfusion measurements from the finger and the chest, on the exterior surface the skin. Previous workers 12, 13, 14, 15 have shown that, even after 9 months and with mean capsule thickness of 2.3 ± 1.8 mm (13), high fidelity signals are still acquired. In the authors’ laboratory, a pilot recording was conducted with a laser Doppler sensor placed inside a mature capsule of a patient needing device extraction, and this showed no impairment of signal fidelity (Online Figure 4).

### Study limitations

This study focused on the VF zone because, if these rates persist, ICDs are generally programmed to deliver a shock. T-wave oversensing, lead fracture, and electromagnetic interference readily generate rates in the VF zone and thereby cause inappropriate shocks.

The present study relied on VF induced in patients under sedation or general anesthesia rather than spontaneous VF. Patients were lying still in a controlled environment and not in the community where most VF and shock events occur. We do not know whether the reliability of the electromechanical coupling approach would still be high outside our controlled research environment. However, a higher performance than with conventional algorithms were observed and a considerable margin of safety. In the present study, laser Doppler recordings were made noninvasively, the running mean and oscillatory height methods could suffer from artifacts that may be inherent in this approach. Further studies using invasive recordings are now required to confirm whether the promising results for the electromechanical coupling method also apply to invasive perfusion recordings.

A previous study (12) using the running mean method and near infrared spectroscopy found that with longer detection windows (15 s), sensitivity for VF detection was improved. In the current study, only standard duration clinical ICD VF detection windows were used. If VF is allowed to continue for sufficient time, eventually almost any method of processing will diagnose it. However, in clinical practice, promptness of diagnosis of VF is considered desirable.

EGM and marker channel data were unable to be extracted from some patients, and for them surface ECG data were used instead. Reassuringly, despite this, the performance of the electromechanical coupling algorithm remained good.

This study addressed VF and its imitators because there is a binary gold standard: it is always appropriate to shock VF and inappropriate to shock its imitators. It is therefore possible to determine indisputably whether an algorithm is performing correctly in each case. The authors’ laboratory has now embarked on a study of VT and SVT, which is different because it can be less obvious whether device intervention is necessary, even if the electrical rhythm is correctly diagnosed.

## Conclusions

Strong electromechanical coupling is a reliable discriminator between VF and situations that mimic VF, achieving an AUC of ROC curve of 1.00 (100% sensitivity and 100% specificity).

Because there is such a large margin of safety between the electromechanical coupling scores of VF and situations that mimic VF, the discrimination is successful even with no personalization to an individual patient. This means that a hemodynamic sensor would only need to be switched on during suspected VF. With electromechanical coupling quantification, the hemodynamic guidance is reliable enough to be used by an ICD to withhold inappropriate shocks. Incorporating such methods into future ICDs may permit reductions of inappropriate shocks whilst preserving safety.

Perspectives **COMPETENCY IN MEDICAL KNOWLEDGE:** ICDs remain limited by inappropriate therapy delivery and are associated with psychological harm and increased morbidity and even mortality. Attempts to incorporate a measure of real-time hemodynamic status into ICDs have previously been attempted. Issues with battery drain and signal analysis have stopped prior progress. Hemodynamic signals are susceptible to noise, artifact, baseline, and biological variability and therefore need to be analyzed in a way that mitigates these effects. A novel algorithm exists that can accurately differentiate clinical status during ventricular fibrillation from sinus rhythm and from artifacts that commonly cause inappropriate ICD therapies.

**TRANSLATIONAL OUTLOOK:** In the present study, acute perfusion measurements were made from the skin surface. Positive findings justify further work to assess the reliability of chronic implantation of laser Doppler sensors, for example, to assess the effect of capsule formation and exercise. If the signal, as expected, remains reliable, adequately powered longer-term studies with implantable sensors will be required in an ICD patient population. This would allow the potential impact for this approach to reduce ICD therapies to be assessed. Reductions may be predicted for patients who otherwise receive an ICD therapy without experiencing hemodynamic compromise. This may be when therapies are given either inappropriately (no ventricular arrhythmia) or unnecessarily (well-tolerated ventricular arrhythmia).
