# Supplementary material for: Quantification of Electromechanical Coupling to Prevent Inappropriate Implantable Cardioverter-Defibrillator Shocks
Source: JACC Clin Electrophysiol. 2019 Jun;5(6):705–15. doi: 10.1016/j.jacep.2019.01.025 (PMC6597902; doi:10.1016/j.jacep.2019.01.025)
Supplement: Online Figures 1–4 [file mmc1.pdf]

Supplementary Material

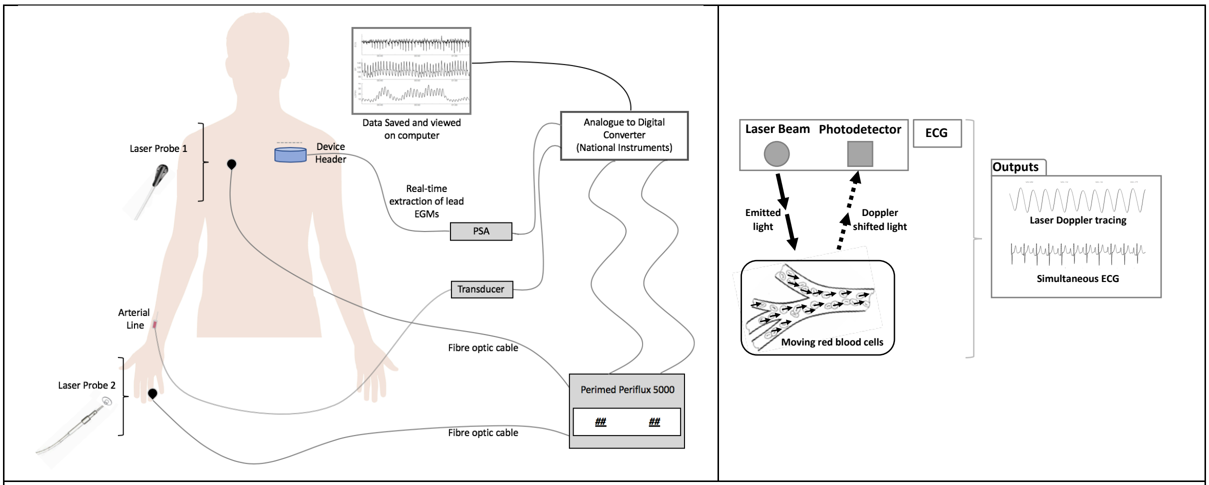

Supplementary Figure 1: Laser Doppler Perfusion Monitoring Basics

Left Panel: Shows the position of the laser Doppler probes. Both were placed on the contralateral side to the implanted device; one on the right chest wall and one on the finger. Signals travel via a fibre optic cable to the Perimed Periflux 5000 device. From here they are converted from analogue to digital signals together with inputs from the ICD and blood pressure. They are then displayed and saved on a PC for analysis.

Right Panel: Moving red blood cells cause a detectable Doppler shift in transmitted laser light. The degree of shift relates to the degree of pulsatile blood flow. A laser Doppler trace is recorded as an oscillatory trace with each oscillation being synchronous with an R wave from simultaneous ECG recording.

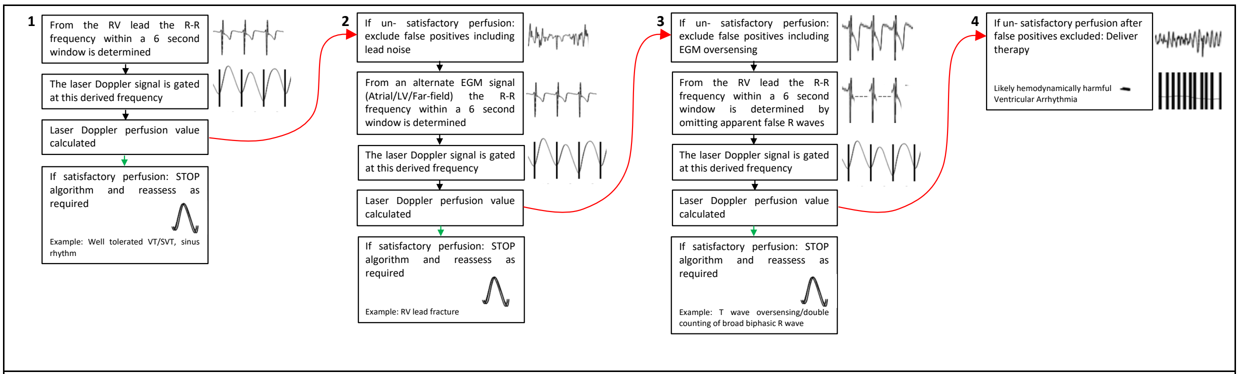

Supplementary Figure 2: Summary of The Electro-Mechanical Coupling Algorithm

Three simultaneous analyses of the laser Doppler data are performed. For ease of understanding here we consider them as if they occur in turn. 1) Gating of the laser Doppler signal is performed based on the R-R interval from the RV defibrillator lead. If satisfactory perfusion is noted the algorithm stops. 2) Gating of the laser Doppler trace is performed based on an alternate EGM signal (atrial / LV lead / from an electrode positioned within the generator itself) to mitigate against potential lead fracture of the RV lead. If satisfactory perfusion is noted the algorithm stops. 3) Gating of the laser Doppler signal is performed based on generating multiple hypotheses as to which of the observed R-R intervals are true and testing them

against the perfusion signal. This is to mitigate against potential oversensing of cardiac electrical signals. If satisfactory perfusion is noted the algorithm stops. 4) If unsatisfactory perfusion remains after exclusion of false positives the algorithm determines that haemodynamic harm is occurring and in keeping with the EGM based algorithm would recommend therapy delivery.

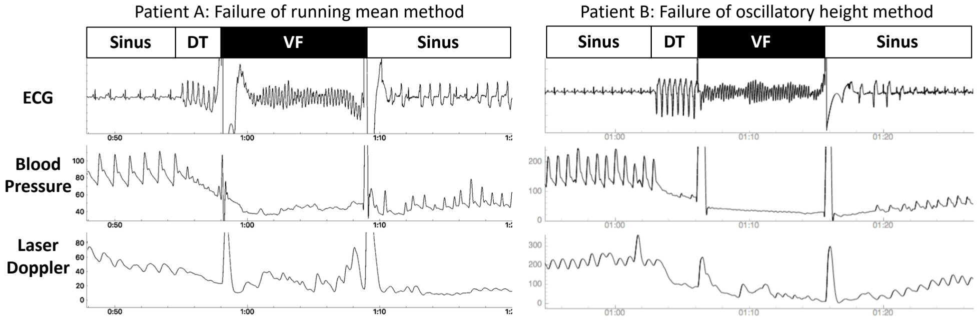

Supplementary Figure 3: Example of Failure of oscillatory height and running mean

Patient A. The Running Mean method fails in this patient as the laser doppler signal does not significantly fall during VF.

Patient B. The Oscillatory Height method fails in this patient as there are still oscillations during the period of VF.

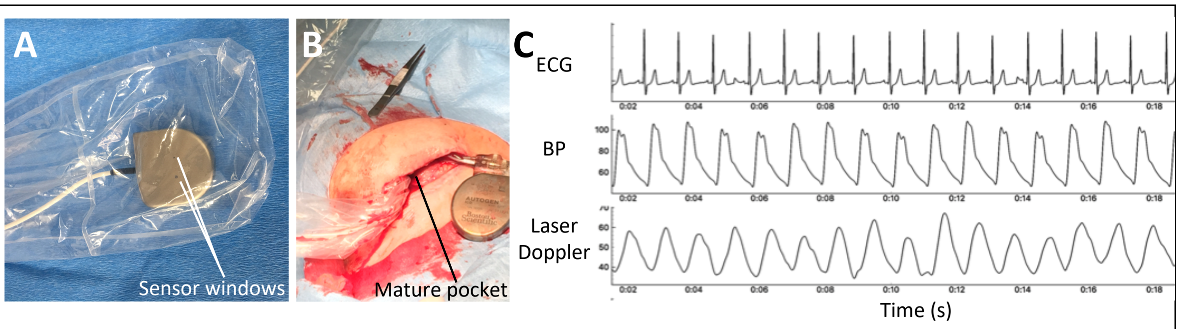

Supplementary Figure 4: Pilot data of signals acquired from mature device pocket.

A: Laser Doppler sensor encased in ICD can with fibre optic cables for data transmission seen extending away from can. Device is placed within sterile sleeve.

B: Laser Doppler device placed within mature pocket.

C: Example data trace showing ECG and BP data (not obtained from can) and simultaneous laser Doppler data occurring in synchronous fashion.
